# The *Lactobacillus *flora in vagina and rectum of fertile and postmenopausal healthy Swedish women

**DOI:** 10.1186/1472-6874-11-17

**Published:** 2011-05-25

**Authors:** Rita J Gustafsson, Siv Ahrné, Bengt Jeppsson, Cecilia Benoni, Crister Olsson, Martin Stjernquist, Bodil Ohlsson

**Affiliations:** 1 Department of Clinical Sciences, Division of Gastroenterology and Hepatology, Skåne University Hospital, °Malmö, Lund University, Lund, Sweden; 2 Applied Nutrition, Lund University, Lund, Sweden; 3 Department of Surgery, Skåne University Hospital, °Malmö, Lund University, Lund, Sweden; 4 Department of Gynecology and Obstetrics, Skåne University Hospital, °Malmö, Lund University, Lund Sweden

## Abstract

**Background:**

*Lactobacillus *species are the most often found inhabitants of vaginal ecosystem of fertile women. In postmenopausal women with low oestrogen levels, *Lactobacillus *flora is diminishing or absent. However, no studies have been performed to investigate the correlation between oestrogen levels and the lactobacilli in the gut. The aim of the present study was to investigate the relation in healthy women between vaginal and rectal microbial flora as well as possible variations with hormone levels.

**Methods:**

Vaginal and rectal smears were taken from 20 healthy fertile women, average 40 years (range 28-49 years), in two different phases of the menstrual cycle, and from 20 postmenopausal women, average 60 years (range 52-85 years). Serum sex hormone levels were analyzed. Bacteria from the smears isolated on Rogosa Agar were grouped by Randomly Amplified Polymorphic DNA and identified by multiplex PCR and partial 16S rRNA gene sequencing.

**Results:**

*Lactobacillus crispatus *was more often found in the vaginal flora of fertile women than in that of postmenopausal (p = 0.036). Fifteen of 20 fertile women had lactobacilli in their rectal smears compared to 10 postmenopausal women (p = 0.071). There was no correlation between the number of bacteria in vagina and rectum, or between the number of bacteria and hormonal levels. Neither could any association between the presence of rectal lactobacilli and hormonal levels be found.

**Conclusion:**

*Lactobacillus crispatus *was more prevalent in the vaginal flora of fertile women, whereas the *Lactobacillus *flora of rectum did not correlate to the vaginal flora nor to hormonal levels.

## Background

Lactobacilli are non-spore-forming, gram-positive rods that form an important part of the normal human bacterial flora commonly found in the mucosa of the mouth [1-3], gastrointestinal (GI) tract [1-4] and female genitourinary tract [1,2,5,6]. They are considered protective organisms required to maintain health by producing lactic acid and other metabolites inhibiting growth of pathogenic organisms [7].

The relationship between vaginal microbial flora, menstruation and levels of oestrogen is complex [8]. The most drastic changes in the vaginal flora occur at the onset of puberty, and are related to an increase of glycogen amount. The elevated glycogen level stimulates the growth of lactic acid-producing bacteria, especially *Lactobacillus *and *Streptococcus *[9]. Those conditions are usually maintained during the fertile years in a healthy vagina and start to change during the menopause. The oestrogen level in fertile women is believed to change during menstrual cycle, and the recovery of the *Lactobacillus *varies slightly [8]. The oestrogen level seems to be a determining factor for colonization of lactobacilli although there is still not any convincing data [10].

Postmenopausal women, who suffer from depletion in vaginal lactobacilli, are sometimes colonized by adverse microbial flora that may cause urinary tract infection [11] and bacterial vaginosis [12]. Nothing is known about the changes in rectal microbial flora in relation to hormonal changes, but some GI diseases tend to have their first onset during the years of the menopause [13,14]. Therefore, our hypothesis was that changes in rectal microbial flora may be an important etiological factor in these entities.

The aim of the present study was to investigate the relationship in healthy women between vaginal and rectal *Lactobacillus *flora, as well as possible variations with sex hormone levels with special references to changes in menstrual phases as well as in the menopause.

## Methods

The study was approved by the Ethics Committee at Lund University. The women gave written informed consent before entering the study.

### Study Design

Twenty healthy fertile women (28-49 years) average 40 years, in two different phases of the menstrual cycle (day 7 and day 21), and 20 healthy postmenopausal women (52-85 years) average 60 years, took part in the study. A basic clinical examination including routine blood samples was performed in the women to ensure a healthy status. Furthermore, a gynecological examination was carried out, including a PAP-smear. The bacterial flora in vagina was normal, excluding bacterial vaginosis or any other infection. All answered a written questionnaire regarding the intake of products containing lactobacilli. The fertile women were asked not to alter their use of hygienic products between the two occasions. All women were asked to report present or single use of drugs for example proton pump inhibitors (PPI), non-steroidal anti inflammatory drugs (NSAID) and antibiotics. The use of any hormonal contraceptive methods or oestrogen hormone replacement therapy was not allowed. Blood samples were collected, centrifuged and serum were stored at -20°C until analyze. Smears from vagina and from rectum were collected with a cotton-tipped swab that were placed in transport medium, on ice and, without delay, transported for cultivation of lactobacilli.

### Cultivation and identification of lactobacilli

Vaginal and rectal smears were treated in an ultrasonic bath for two minutes and diluted before plating on Rogosa agar plates and incubated anaerobically at 37° C for 72 hours. Two to three colonies were randomly picked and characterized by Randomly Amplified Polymorphic DNA (RAPD) as described by Quednau et al. [15], and those having the same RAPD pattern within the same sample were regarded as belonging to the same species. Species identification was performed by multiplex PCR as described by Song et al. [16], slightly modified by Vasquez et al. [17], or if not applicable by partial 16S rDNA sequencing. The multiplex-PCR identified isolates of the following species: *L*. *crispatus*, *L*. *acidophilus*, *L*. *gasseri*, *L*. *jensenii*, *L*. *reuteri*, *L*. *delbrueckii*, *L*. *plantarum*, *L*. *salivarius *and *L*. *paracasei*. Isolates (altogether 13) of *L*. *vaginalis*, *L*. *colehominis *and *L*. *ruminis *were identified by sequencing of approximately 800 bp long fragments of the 16S rRNA gene.

### Sex hormone analyses

Serum oestradiol, progesterone, follicle-stimulating hormone (FSH) and luteinizing hormone (LH) were analyzed for the fertile women at day 7 and 21 and for the postmenopausal women at the Department of Clinical Chemistry, Skåne University Hospital, Malmö. Oestradiol and progesterone were analyzed by a one-step competitive immunoassay with alkalic phosphatase (ALP), enzyme marking and magnetic separation. FSH and LH were analyzed by a two-step immunometric assay with ALP, enzyme marking and magnetic separation.

### Statistical analyses

Fisher´s exact test was used to compare fertile women between day 7 and 21, and fertile and postmenopausal women, in the presence of specific lactobacilli in vagina and rectum. Spearman´s test was used to examine correlations between the number of bacteria in vagina and rectum, and between the number of bacteria and hormonal levels. P < 0.05 was considered statistical significance.

## Results

### Vaginal smears

The colony forming units (CFU) bacteria per gram vaginal smear varied in fertile women from 8.3x10^4^-1.8 × 10^8 ^at day 7, and from 4.0 × 10^2^-4.0 × 10^7 ^at day 21 (Table 1 and 2). In the postmenopausal women it varied from 1.7 × 10^2^-3.0 × 10^7 ^(Table 3).

**Table 1:** Serum hormone levels and bacterial smear numbers in fertile women at day 7 of the menstrual cycle

Subject No.	Oestradiol (E2) (pmol/L)	Progesterone (nmol/L)	FSH (IU/L)	LH (IU/L)	Vaginal smear (CFU/g)	Rectal smear (CFU/g)
1	689	3	4.6	3.9	*--*	180

2	737	< 3	7.7	4.5	1600000	840000

3	653	5	18.2	24.7	98000	950000

4	138	< 3	9.5	5.6	*--*	3200000

5	310	3	8.5	4.4	180000000	230000

6	238	< 3	7.2	3.5	*--*	850000

7	1053	3	6.7	4.3	4200000	350000

8	240	3	6.2	3.5	*--*	2800000

9	166	< 3	8.9	6.5	3000000	190000

10	282	< 3	9.2	2.8	5700000	38000

11	520	< 3	7.1	5.7	7200000	280000

12					*--*	130000

13	1111	3	5.7	8.5	3600000	1600000

14	< 125	3	51.2	12.9	420000	1200000

15	253	3	5.7	5.8	*--*	*--*

16	929	4	4.8	5.5	150000	4300

17	598	< 3	4.7	8.4	83000	180000

18	328	6	7.8	7.1	*--*	*--*

19	150	3	8.9	9.5	*--*	19000000

20	189	3	52.7	19.8	*--*	*--*

*-- *= not detectable. One woman did not complete the hormone analyses. CFU = colony forming units, IU = international units, FSH = follicle-stimulating hormone, LH = luteinizing hormone.

**Table 2:** Serum hormone levels and bacterial smear numbers in fertile women at day 21 of the menstrual cycle

Subject No.	Oestradiol (E2) (pmol/L)	Progesterone (nmol/L)	FSH (IU/L)	LH (IU/L)	Vaginal smear (CFU/g)	Rectal smear (CFU/g)
1	318	52	5.9	2.2	*--*	3000000

2	656	46	4.8	5.8	25000000	79000

3	272	12	2.9	1.4	83000	90000

4	248	35	4.9	4.6	*--*	4000000

5	499	52	2.9	2.2	26000000	1800000

6	253	18	3.1	< 1.0	400	31000

7	374	3	8.0	2.8	40000000	120000

8	274	14	2.6	2.4	*--*	4800000

9	158	< 3	11.9	20.2	22000000	620000

10	389	21	3.1	1.4	8700000	35000

11	635	31	3.8	4.9	3000000	5500

12					*--*	*--*

13	221	18	8.3	3.1	55000	1800

14	705	8	25.2	19.8	710000	1700000

15	326	35	6.0	7.2	17000	510000

16			4.1	3.1	150000	2200

17	164	15	4.8	2.5	1200	380000

18	607	48	5.0	8.4	*--*	21000

19	568	70	3.1	3.0	*--*	*-- *

20	1248	5	2.9	3.5	*--*	3100

*-- *= not detectable. One woman did not complete the hormone analyses and one women only completed FSH and LH analyses. CFU = colony forming units, IU = international units, FSH = follicle-stimulating hormone, LH = luteinizing hormone.

**Table 3:** Serum hormone levels and bacterial smear numbers in postmenopausal women

Subject No.	Oestradiol (E2) (pmol/L)	Progesterone (nmol/L)	FSH (IU/L)	LH (IU/L)	Vaginal smear (CFU/g)	Rectal smear (CFU/g)
21	< 125	< 3	66.3	36.5	*--*	1100000

22	126	< 3	33.7	16.3	170	5500

23	< 125	< 3	65.4	24.6	10000000	3100000

24	133	< 3	84.2	31.4	*--*	3000000

25	193	< 3	58.6	21.0	*--*	3800000

26	< 125	< 3	58.8	26.7	360000	140000

27	< 125	< 3	73.5	28.9	13000	590000

28	< 125	< 3	145.0	50.9	*--*	1700000

29	< 125	< 3	80.9	16.2	830000	47000

30	< 125	< 3	48.7	15.7	180000	4900000

31	< 125	< 3	65.1	33.4	*--*	1000

32					*--*	130000

33	< 125	< 3	50.8	37.8	8900000	*--*

34	< 125	3	89.6	39.9	510000	50000

35	< 125	3	126.0	53.7	30000000	920000

36	< 125	< 3	88.1	43.8	22000	210000

37	< 125	< 3	53.3	19.0	*--*	910000

38	< 125	< 3	89.7	23.1	*--*	550000

39	< 125	< 3	110.0	38.2	190000	3100

40	< 125	< 3	165.0	53.8	*--*	2000000

*-- *= not detectable. One woman did not complete the hormone analyses. CFU = colony forming units, IU = international units, FSH = follicle-stimulating hormone, LH = luteinizing hormone.

**Figure 1:**
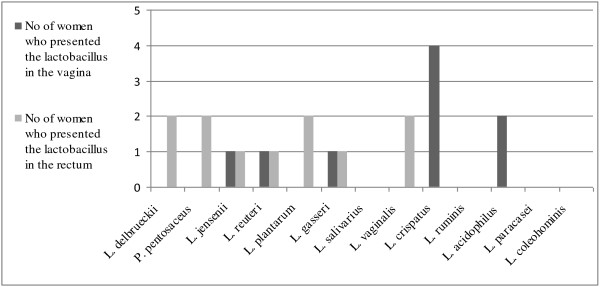
**Presence of *Lactobacillus *species in fertile women at day 7 in the vagina and rectum**.

**Figure 2:**
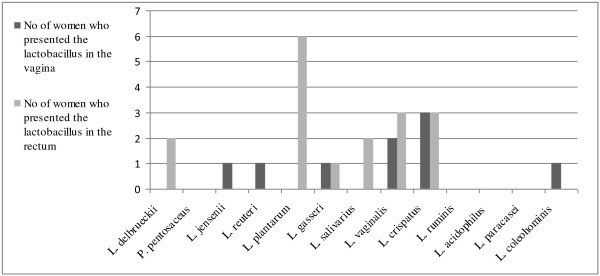
**Presence of *Lactobacillus *species in fertile women at day 21 in the vagina and rectum**.

**Figure 3:**
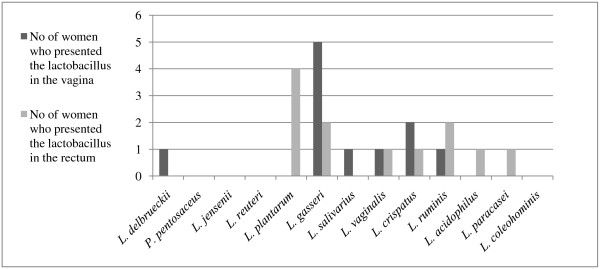
**Presence of *Lactobacillus *species in postmenopausal women in the vagina and rectum**.

Lactobacilli were found and isolated from 11 out of 20 fertile women as well as from 11 out of 20 menopausal women (Table 4 and 5). Altogether 39 isolates were further characterized. The most commonly identified species of the fertile women was *L. crispatus *(7 women), followed by *L. acidophilus *(2 women)*, L gasseri *(2 women)*, L. jensenii *(2 women) and *L. vaginalis *(2 women) (Figure 1 and 2). Menopausal women were most often colonized by strains of *L. gasseri *(5 women) and *L. crispatus *(2 women) (Figure 3). *Lactobacillus crispatus *was significantly more often found in the vaginal flora of the fertile women than in that of postmenopausal women (p = 0.036).

**Table 4:** Presence of different *Lactobacillus *species in the vagina and rectum in fertile women

Subject No.	Day in menstrual cycle	Vaginal smear	Rectal smear
1	7	*--*	*L. plantarum*

2	7	*L. crispatus*	*L. delbrueckii*
	
	21	*L. crispatus*	*L.vaginalis*, *L. delbrueckii*

3	21	*L. coleohominis*, *L. vaginalis*	*L. vaginalis*

4	7	*--*	*L. vaginalis*

5	7	*L. crispatus*	*L. plantarum*

	21	*L. crispatus*	*L. vaginalis*

6	7	*--*	*L. vaginalis*
	
	21	*--*	*L. plantarum*

7	7	*L. crispatus*	*L. delbrueckii*
	
	21	*L. crispatus*	*L. crispatus*, *L. delbrueckii*

9	7	*L. acidophilus*	*--*
	
	21	*L. acidophilus*	*--*

10	7	*L. acidophilus*	*L. plantarum*, *L. crispatus*
	
	21	*L. acidophilus*, *L. crispatus*	*--*

12	7	*L.gasseri*	*L.gasseri*
	
	21	*L.gasseri*	*L.gasseri*, *L. salivarius*

13	7	*L. jensenii, L. reuteri*	*L. jensenii, L. reuteri*
	
	21	*L. jensenii, L. reuteri*	*L. salivarius*

14	7	*L. crispatus*	*--*
	
	21	*L.vaginalis*	*L. plantarum*

15	21	*L. crispatus*	*L. plantarum*

16	7	*L. jensenii*	*--*
	
	21	*L. crispatus*	*L. crispatus*

18	21	*--*	*L. plantarum*

19	21	*--*	*L. plantarum*

*-- *= not detectable. Deleted subjects or lines mean that no lactobacilli were found.

**Table 5:** Presence of different *Lactobacillus *species in the vagina and rectum in postmenopausal women

Subject No.	Vaginal smear	Rectal smear
21	*--*	*L. acidophilus*, *L. plantarum*

22	*L. gasseri*	*L. gasseri*

23	*L. crispatus*	*L. plantarum*

26	*L. salivarius*	*--*

27	*L. delbrueckii*	*--*

29	*L. vaginalis*	*L. plantarum, L. gasseri*

30	*L. gasseri*	*L. vaginalis*

32	*--*	*L. crispatus*

33	*L. crispatus*	*--*

34	*L. gasseri*	*L. ruminis*

35	*L. gasseri*	*L. plantarum*

36	*L. ruminis*	*L. ruminis*

39	*L. gasseri*	*L. paracasei*

*-- *= not detectable. Deleted subjects or lines mean that no lactobacilli were found.

### Rectal smears

The CFU bacteria per gram rectal smear of the fertile group varied between 1.8 × 10^2^-1.9 × 10^7 ^at day 7, and between 2.2 × 10^3^-4.8 × 10^6 ^at day 21 (Table 1 and 2). In postmenopausal women, the CFU/g varied between 1.0 × 10^3^-4.9 × 10^6 ^(Table 3).

Lactobacilli were found and isolated from 15 out of 20 fertile women as compared to 10 out of 20 menopausal women (p = 0,071) (Table 4 and 5). Altogether 67 isolates were further characterized. The most often occurring *Lactobacillus *species in the rectal smear of fertile women was *L. plantarum *(8 women), followed by *L. vaginalis *(5 women), *L. crispatus *(3 women), *L. delbrueckii *(2 women) and *L. salivarius *(2 women) (Figure 1 and 2). Also in postmenopausal women, the most commonly found lactobacilli was the species *L. plantarum *(4 women), followed by *L. gasseri *(2 women) and *L.ruminis *(2 women) (Figure 3).

### Correlation between vaginal and rectal smears

Eight women had the same *Lactobacillus *species in their vaginal and rectal smears, six in the fertile group and two in the menopausal group (Table 4 and 5). This difference was not statistically significant. The number of bacteria in vaginal and rectal smears within a woman was not correlated (data not shown).

Seven fertile women were in the vagina colonized by the same *Lactobacillus *species on day 7 and 21 of the menstrual cycle. Three of those were also colonized by that species in the rectum. The remaining were, both in vagina and rectum, colonized by different species during those days (Table 4).

In two menopausal women, the same species of lactobacilli (*L. gasseri *and *L. ruminis*) was found to dominate in both vaginal and rectal smears (Table 5).

There was no statistically significant fluctuation of the number of bacteria in the rectal flora between day 7 and day 21 of the menstrual cycle, nor between fertile and postmenopausal women (data not shown).

### Sex hormone levels in serum

Oestrodiol levels were lower than125 pmol/L for most of the postmenopausal women (Table 3). In the fertile women it varied from < 125-1111 pmol/L at day 7, and from 158-1248 pmol/L at day 21 (Table 1 and 2). For the fertile women at day 7 and for all the postmenopausal women, progesterone levels were equal or lower than 6 nmol/L, and for the fertile women at day 21 the levels varied from < 3-70 nmol/L. FSH levels were 4.6-52.7 IU/L in the fertile women at day 7, and 2.6-25.2 IU/L at day 21. FSH was much higher in the postmenopausal women, 33.7-165.0 IU/L. The same was seen for LH which had lower levels in the fertile women, 2.8-24.7 IU/L at day 7 and < 1.0-20.2 IU/L at day 21, than the postmenopausal women, 15.7-53.8 IU/L (Table 1, 2 and 3). These values were within the normal ranges.

### Correlations between *Lactobacillus *and sex hormone levels

*Lactobacillus crispatus *was present in the vaginal ecosystem in fertile women with high (1053 pmol/L) and medium (310-737 pmol/L) oestradiol levels. This reached statistical significance compared to postmenopausal women (p = 0.036). However, one woman with the lowest oestradiol level ( < 125 pmol/L) was colonized by this species as well. *Lactobacillus jensenii, L. reuteri *and *L. vaginalis *were present in the vaginal ecosystems in women with high and medium oestradiol values. There was no correlation between the number of vaginal bacteria and hormone levels. No associations were found between rectal microbial flora and sex hormone levels (data not shown).

## Discussion

The purpose of this pilot study was to investigate the relationship between vaginal and rectal *Lactobacillus *flora in healthy women, and possible variations with sex hormonal levels. Several studies have shown that the vaginal microbial flora varies in relationship to hormonal levels. However, no studies have been performed to investigate the correlation between hormonal levels and lactobacilli in the gut. Therefore, we analyzed vaginal and rectal smears from fertile and postmenopausal women as well as serum levels of oestradiol, progesterone, FSH and LH. In the fertile women, smears were collected both in day 7 and day 21 of the menstrual cycle.

The main finding in our present study was that *L. crispatus *was more often found in the vaginal flora of fertile women than in postmenopausal women. Previous studies have also demonstrated that *L. crispatus *is more often found in fertile than postmenopausal women [5,17,18].

No relation was found between numbers or occurrence of species of lactobacilli found in the vagina and rectum in a specific woman. However, this was a small study. In a recent large study of 531 fertile women in the age of 14-35 years, 43% of those having *L. crispatus *in vagina also had this species in rectum [19]. These authors found *L. crispatus*, together with *L. jensenii*, to be the most common species in vagina and in rectum. Interestingly, *L. vaginalis *and *L. plantarum *that was found as dominating lactobacilli from rectal smears in our study was not at all identified as part of rectal flora in the study of Antonio et al. [19]. However, the age of our study group was considerably higher (28-49 years) and the methodology of identification of lactobacilli was different.

*Lactobacillus vaginalis *was first described by Embley et al. 1989 [20]. It was isolated from the vagina of patients suffering from trichomoniasis. *Lactobacillus vaginalis *is a hydrogen peroxide (H_2_O_2_)-producing lactobacilli, and is together with *L. crispatus, L. jensenii and L. gasseri *the most commonly recovered species of H_2_O_2_-producing lactobacilli in the vagina [5,21-23]. There are so far not many studies of *L. vaginalis*, but it has been shown that the absence of H_2_O_2_-producing lactobacilli in the vagina is associated with an increase of bacterial vaginosis [24,25]. In a previous study, vaginal and rectal colonization by H_2_O_2_-producing lactobacilli was associated with a lower prevalence of bacterial vaginosis, compared with vaginal colonization alone. This may suggest that rectal colonization by H_2_O_2_-producing lactobacilli may contribute to maintain the vaginal ecosystem [19]. In the present study no women with bacterial vaginosis were included, why comparisons according to presence or absence of different lactobacilli in rectum in this entity could not be performed.

Menopausal and postmenopausal women are at risk for autoimmune as well as metabolic and cardiovascular diseases [26-28]. Different hormonal, immunological and vascular changes might contribute to the increased risk for GI dysfunction in ageing women [13,14]. Our hypothesis that changes in rectal microbial flora may be an important etiological factor in these entities could not be confirmed in the present study.

One of the limitations of our study is the low number of examined women. However, this study was performed as a pilot study, aimed to generate a larger study if any positive results were found. Furthermore, established bacterial growth was found in only half of the smears. The explanation to this may partly be that we did not search for *L. iners*, which differs from other *Lactobacillus *species due to its peculiar culture requirements [29]. It has been shown that *L. iners *is part of the normal vaginal microbial flora [17], but further studies show that *L. iners *is a dominant part of the vaginal microbial flora when it is in a transitional stage between abnormal and normal due to treatment, physiological changes or oestrogen levels [30-32]. The fact that the smears were taken from the distal colon should not influence our results as there is little regional difference in human colonic microbial flora [33].

## Conclusions

In this pilot study, we did not find any correlation between the overall levels of *Lactobacillus *species in vagina and rectum, and variations in sex hormone levels. However, *L. crispatus *was more often found in the vaginal flora of fertile women than in that of postmenopausal women.

## Competing interests

The authors declare that they have no competing interests.

## Authors' contributions

RG designed the study, collected the data, contributed to the statistical analyses and wrote the manuscript together with BO. SA designed the study and performed the cultivation and identification of lactobacilli. BJ financially supported the cultivation and identification of lactobacilli (Bengt Ihre Foundation). CB initiated and designed the study. CO contributed to the statistical analyses. MS designed the study and collected the vaginal and rectal smears. BO financially supported the study (Development Foundations of Region Skane), contributed to the statistical analyses and wrote the manuscript together with RG. All authors contributed to the manuscript with constructive criticism, and read and approved the final manuscript.

## Pre-publication history

The pre-publication history for this paper can be accessed here:

http://www.biomedcentral.com/1472-6874/11/17/prepub
